# Association between glymphatic dysfunction and cryptogenic stroke risk in patients with patent foramen ovale: a retrospective cross-sectional study

**DOI:** 10.3389/fneur.2025.1620772

**Published:** 2025-09-22

**Authors:** Weitao Zhang, Fengfeng Wang, Qun Li

**Affiliations:** Department of Children's Heart Center, Fuwai Central China Cardiovascular Hospital, Zhengzhou University Central China Fuwai Hospital, Zhengzhou, China

**Keywords:** patent foramen ovale, cryptogenic stroke, glymphatic system, ALPS index, diffusion tensor imaging, risk stratification

## Abstract

**Background:**

Patent foramen ovale (PFO) is strongly associated with cryptogenic stroke (CS), but the underlying mechanisms remain incompletely understood. The glymphatic system plays a crucial role in central nervous system homeostasis, and its dysfunction has been implicated in various neurological disorders. This study aimed to evaluate the association between glymphatic dysfunction, assessed by the ALPS (Analysis Along the Perivascular Space) index, and the risk of cryptogenic stroke in patients with PFO.

**Methods:**

This retrospective, single-center cross-sectional study enrolled 208 PFO patients, including 52 with cryptogenic stroke and 156 without a history of stroke. All participants underwent brain MRI with diffusion tensor imaging (DTI) to calculate the ALPS index. Clinical data, laboratory tests, and echocardiographic parameters were collected. Multivariate logistic regression was used to identify independent predictors of cryptogenic stroke, and receiver operating characteristic (ROC) curve analysis was performed to assess the diagnostic performance of the ALPS index.

**Results:**

Patients with cryptogenic stroke exhibited significantly lower ALPS index values compared to controls (1.31 ± 0.18 vs. 1.52 ± 0.21, *p* < 0.001). Multivariate analysis demonstrated that a lower ALPS index (OR = 0.126, 95% CI: 0.059–0.273, *p* < 0.001) was independently associated with cryptogenic stroke after adjusting for confounders. The ALPS index showed excellent diagnostic performance, with an AUC of 0.916 (95% CI: 0.876–0.956), yielding a sensitivity of 90.7% and specificity of 82.2% at the optimal cut-off value.

**Conclusion:**

In PFO patients, impaired glymphatic function, as indicated by a lower ALPS index, was independently associated with an increased risk of cryptogenic stroke. The ALPS index may serve as a promising non-invasive imaging biomarker for stroke risk stratification in this high-risk population.

## Background

Stroke is the second leading cause of global economic burden and one of the major causes of non-traumatic disability (1–3). According to the TOAST classification for ischemic stroke, ~30%−40% of ischemic stroke cases have no identifiable cause and are classified as cryptogenic stroke (CS) (4–6). Incomplete fusion of the septum primum and septum secundum during fetal development can leave a small gap, commonly referred to as a patent foramen ovale (PFO) (7). The prevalence of PFO in the general adult population is estimated to be ~25%−34% (8, 9). Previous studies have reported that more than half of patients with cryptogenic stroke have a coexisting PFO (4, 10), and this proportion increases to 61% among individuals aged over 55 years (11, 12). Furthermore, the presence of PFO is associated with a threefold increased risk of stroke recurrence (7, 13–16). These findings highlight the strong association between cryptogenic stroke and PFO, underscoring the critical importance of preventing stroke recurrence in this high-risk population (4, 17).

Cerebrospinal fluid (CSF) within the subarachnoid space enters the brain parenchyma through perivascular spaces, where it exchanges with interstitial fluid (18, 19). This process plays a critical role in maintaining central nervous system (CNS) homeostasis, functioning similarly to a lymphatic system (20, 21). The glymphatic system facilitates the distribution and transport of glucose, macromolecules, electrolytes, and pharmacological agents injected into the CSF throughout the brain (22–24). Moreover, it is increasingly recognized as being involved in the pathophysiological processes and functional recovery of various neurological disorders, including stroke, Alzheimer's disease, and traumatic brain injury (25–27). In addition, an increasing number of studies have utilized diffusion tensor imaging analysis along the perivascular space (DTI-ALPS) as a non-invasive method to assess glymphatic system function in the brain (28–31).

Previous studies have suggested that patients with PFO may experience states of hypoxia and hypoperfusion, which could increase the burden of enlarged perivascular spaces (EPVS) and contribute to glymphatic dysfunction in this population (32, 33). However, despite increasing recognition of the glymphatic system's role in neurological diseases, little is known about the relationship between glymphatic dysfunction and cryptogenic stroke in patients with PFO. We hypothesized that glymphatic system dysfunction, as reflected by a lower ALPS (Analysis Along the Perivascular Space) index, is associated with an increased risk of cryptogenic stroke in patients with PFO. The objective of this study was to evaluate the association between ALPS index and cryptogenic stroke risk in patients with PFO. This work aims to improve understanding of how cardiac structural anomalies relate to glymphatic dysfunction and to explore the potential of ALPS index as a biomarker for stroke risk stratification.

## Methods

### Study design and population

This retrospective, single-center cross-sectional study was conducted at Fuwai Hospital, Zhengzhou University between January 2020 and December 2023. We consecutively enrolled 208 patients with PFO confirmed by contrast TEE (agitated saline with Valsalva maneuver). Participants were divided into two groups: (1) Cryptogenic stroke group (*n* = 52), diagnosed as embolic stroke of undetermined source (ESUS) according to 2014 AHA/ASA criteria after excluding other etiologies; and (2) Control group (*n* = 156), consisting of PFO patients without a history of stroke, enrolled during the same study period. Although demographic balance was considered during recruitment, no formal matching procedures were applied, and observed differences in age and sex were accounted for in the multivariate analyses.

The study protocol was approved by the Institutional Review Board of Fuwai Hospital. All participants provided written informed consent.

### Inclusion criteria and exclusion criteria

Inclusion criteria: (1) PFO with right-to-left shunt; (2) Complete workup (MRI, vascular imaging, echocardiography, thrombophilia tests). Exclusion criteria: (1) Stroke of determined etiology (TOAST classification); (2) Active neurological diseases (e.g., epilepsy, neurodegenerative disorders); (3) Contraindications to MRI, including the presence of metallic implants or a glomerular filtration rate (GFR) < 30 mL/min/1.73 m^2^; (4) Poor imaging quality precluding reliable analysis.

### Definition and assessment of cryptogenic stroke

Cryptogenic stroke was defined according to the 2014 American Heart Association/American Stroke Association criteria for embolic stroke of undetermined source after excluding: (1) Atherosclerosis: < 50% stenosis in arteries supplying the infarct; (2) Cardio-embolism: no atrial fibrillation (≥24-h Holter monitoring) or other major sources (TTE/TEE-confirmed); (3) Small vessel disease: no lacunar infarcts (≤1.5 cm with classic clinical-radiologic correlation) (34, 35).

All patients underwent: (1) Brain MRI (DWI/FLAIR sequences to confirm acute infarction); (2) Vascular imaging: MRA/CTA of cervical and intracranial vessels; (3) Cardiac monitoring: ≥24-h Holter with arrhythmia detection threshold set at ≥30 sec; (4) Echocardiography: TTE to exclude structural abnormalities; TEE with bubble study (agitated saline + Valsalva) to grade PFO shunt (ISAC classification) and measure ISA (excursion ≥10 mm).

### Laboratory measurements

Laboratory parameters were collected from fasting blood samples and included hemoglobin levels, platelet count, neutrophil count, lymphocyte count, fasting glucose, lipid profile (total cholesterol, LDL, HDL, and triglycerides), and estimated glomerular filtration rate (GFR). Behavioral factors, such as current smoking (defined as smoking at least one cigarette per day within the past 30 days) and alcohol consumption, were also documented.

### Transthoracic echocardiography (TTE)

All participants underwent standardized transthoracic echocardiography (TTE). Left ventricular ejection fraction (LVEF) was measured using the biplane Simpson's method from apical four-chamber and two-chamber views (36, 37). Right ventricular systolic pressure was estimated from the peak tricuspid regurgitation jet velocity using the Bernoulli equation.

### Contrast-enhanced transesophageal echocardiography (TEE)

Contrast-enhanced transesophageal echocardiography (TEE) was performed using a Philips EPIQ 7C ultrasound system equipped with an X7–2t transducer. A contrast study was conducted by injecting 10 mL of agitated saline during a standardized Valsalva maneuver. The right-to-left shunt was graded according to International Consensus criteria: Grade 1 (< 10 microbubbles), Grade 2 (10–30 microbubbles), and Grade 3 (>30 microbubbles observed in the left atrium) (38, 39). Morphological features assessed included maximal PFO tunnel diameter (measured at end-diastole in the bicaval view), PFO tunnel length (measured as the septal overlap distance at 90° view), and the presence of interatrial septal aneurysm (ISA), defined as an interatrial septal excursion ≥10 mm during the cardiac cycle (40, 41).

### Assessment of ALPS index

All participants underwent brain magnetic resonance imaging (MRI) on a 3.0 Tesla system using a 64-channel phased-array head coil. Diffusion tensor imaging (DTI) data were acquired using a single-shot spin-echo echo-planar imaging (EPI) sequence. The imaging parameters were as follows: b-values of 0 and 1,000 s/mm^2^, 64 diffusion-encoding directions, repetition time (TR) of 8,000 ms, echo time (TE) of 85 ms, voxel resolution of 2 × 2 × 2 mm3, and total acquisition time of ~8 min and 32 s. Image preprocessing and ALPS index calculation were performed using the FMRIB Software Library (FSL, version 6.0). Preprocessing steps included eddy current correction, motion artifact removal, and skull stripping using the Brain Extraction Tool (BET). Regions of interest (ROIs) were manually placed on fractional anisotropy (FA) maps by two experienced neuroradiologists blinded to clinical data. The ROIs targeted projection fibers (PF) located along the lateral ventricular body and association fibers (AF) located superior to the PF in the corona radiata. Interobserver agreement was excellent, with an intraclass correlation coefficient (ICC) of 0.89 for ROI placement. The ALPS index was calculated by summing the diffusivity along the x-axis (Dxx) in the projection and association fiber ROIs and dividing this by the sum of the diffusivity along the y-axis in the projection fibers (Dyy) and the diffusivity along the z-axis in the association fibers (Dzz), with higher ALPS index values indicating better glymphatic system function.

### Statistical analysis

All statistical analyses were performed using SPSS version 26.0 (IBM Corp., Armonk, NY, USA). Continuous variables were expressed as mean ± standard deviation (SD) and compared between groups using independent-sample *t*-tests. Categorical variables were presented as counts and percentages, and compared using the chi-square test or Fisher's exact test, as appropriate. Univariate logistic regression analyses were conducted to identify potential factors associated with cryptogenic stroke. Variables with a *p*-value < 0.05 in univariate analyses were further included in multivariate logistic regression models to identify independent predictors. Odds ratios (ORs) with 95% confidence intervals (CIs) were reported. The associations between ALPS index and echocardiographic parameters were assessed using linear regression models. Three models were constructed: Model 1 was unadjusted, Model 2 was adjusted for age, sex, and body mass index (BMI), and Model 3 was additionally adjusted for systolic blood pressure, diastolic blood pressure, and years of education. The diagnostic performance of the ALPS index in predicting cryptogenic stroke was evaluated using receiver operating characteristic (ROC) curve analysis. The area under the curve (AUC) with 95% CI was calculated, and the optimal cut-off value was determined based on the Youden index. Sensitivity and stratified analyses. To address potential residual age confounding, we performed age-restricted sensitivity analyses limited to participants aged ≤ 55 years (primary) and ≤ 60 years (secondary), refitting multivariable logistic regression models using the same covariate set as in the main analysis. To explore effect modification by right-to-left shunt burden and interatrial septal aneurysm (ISA), we conducted stratified analyses by shunt grade (ISAC Grades 1–3) and by ISA status (present vs absent), reporting stratum-specific odds ratios (ORs) and 95% confidence intervals (CIs). Interaction was tested by including product terms (ALPS × shunt grade; ALPS × ISA) and by likelihood-ratio tests comparing models with and without interaction terms. A *p*-value < 0.05 was considered statistically significant.

## Results

### Baseline characteristics

A total of 208 patients with PFO were included in the analysis, comprising 156 individuals without stroke (control group) and 52 patients with cryptogenic stroke. As shown in Table 1, patients with cryptogenic stroke were significantly older than those in the control group (53.61 ± 14.32 vs. 48.23 ± 16.14 years, *p* = 0.021). The proportion of male participants was higher in the stroke group (59.62%) compared to controls (43.59%), with a statistically significant difference (*p* = 0.038). Furthermore, the prevalence of hypertension (48.08% vs. 32.69%, *p* = 0.047), hyperlipidemia (30.77% vs. 18.59%, *p* = 0.049), and current smoking (40.38% vs. 28.85%, *p* = 0.012) was significantly greater in the cryptogenic stroke group. There were no significant differences between the groups in the prevalence of diabetes mellitus (*p* = 0.118) or coronary artery disease (CAD) (*p* = 0.342).

**Table 1:** Baseline characteristics of study participants.

**Variable**	**Control group (*n* = 156)**	**Cryptogenic stroke (*n* = 52)**	***p-value***
Age (years), mean ± SD	48.23 ± 16.14	53.61 ± 14.32	0.021^*^
Male gender, *n* (%)	68 (43.59%)	31 (59.62%)	0.038^*^
Hypertension, *n* (%)	51 (32.69%)	25 (48.08%)	0.047^*^
Diabetes mellitus, *n* (%)	22 (14.10%)	12 (23.08%)	0.118
Hyperlipidemia, *n* (%)	29 (18.59%)	16 (30.77%)	0.049^*^
CAD, *n* (%)	19 (12.18%)	9 (17.31%)	0.342
Current smoking, *n* (%)	45 (28.85%)	21 (40.38%)	0.012^*^

Data are presented as mean ± standard deviation (SD) or number (%). ^*^p < 0.05 indicates statistical significance.

CAD, coronary artery disease.

### Echocardiographic characteristics

Echocardiographic parameters were compared between the control group and patients with cryptogenic stroke, all of whom had PFO. As shown in Table 2, the incidence of interatrial septal aneurysm (ISA) was significantly higher in the stroke group compared to controls (55.77% vs. 30.13%, *p* < 0.001). In contrast, the presence of a prominent Eustachian valve did not differ significantly between the groups (9.62% vs. 5.77%, *p* = 0.342). The PFO tunnel diameter was significantly larger in patients with cryptogenic stroke (2.31 ± 1.05 mm vs. 1.82 ± 0.97 mm, *p* = 0.003), while no significant difference was observed in PFO tunnel length (11.24 ± 3.45 mm vs. 10.87 ± 3.92 mm, *p* = 0.514). Left ventricular ejection fraction (LV EF) was comparable between the groups (58.76 ± 4.91% vs. 57.89 ± 5.83%, *p* = 0.275).

**Table 2:** Echocardiographic characteristics of study participants.

**Variable**	**Control group (*n* = 156)**	**Cryptogenic stroke (*n* = 52)**	***p-value***
ISA, *n* (%)	47 (30.13%)	29 (55.77%)	< 0.001^*^
Prominent Eustachian valve, *n* (%)	9 (5.77%)	5 (9.62%)	0.342
PFO tunnel length (mm), mean ± SD	10.87 ± 3.92	11.24 ± 3.45	0.514
PFO tunnel diameter (mm), mean ± SD	1.82 ± 0.97	2.31 ± 1.05	0.003^*^
LV EF (%), mean ± SD	57.89 ± 5.83	58.76 ± 4.91	0.275

ISA, interatrial septal aneurysm; LV EF, left ventricular ejection fraction. ^*^p < 0.05 indicates statistical significance.

### Laboratory and imaging biomarkers

The laboratory and imaging biomarkers of PFO patients were compared between the control and cryptogenic stroke groups (Table 3). There were no significant differences between the groups regarding hemoglobin, platelet count, neutrophil count, lymphocyte count, glucose level, or LDL cholesterol (all *P* >0.05). However, patients with cryptogenic stroke exhibited significantly higher total cholesterol levels (203.18 ± 48.57 vs. 186.75 ± 46.32 mg/dL, *p* = 0.009) and triglyceride levels (161.82 ± 72.95 vs. 131.46 ± 68.37 mg/dL, *p* < 0.001), along with lower HDL cholesterol levels (46.83 ± 12.64 vs. 51.27 ± 13.95 mg/dL, *p* = 0.023), compared to controls. Additionally, the glomerular filtration rate (GFR) was slightly higher in the stroke group (93.15 ± 19.82 vs. 86.73 ± 22.45 mL/min/1.73 m^2^, *p* = 0.028). Importantly, the ROPE score, an indicator of the likelihood of a stroke being related to a PFO, was significantly higher in the cryptogenic stroke group (6.39 ± 1.81 vs. 4.82 ± 2.07, *p* < 0.001). Furthermore, the ALPS index, reflecting glymphatic system function, was significantly lower in patients with cryptogenic stroke compared to controls (1.31 ± 0.18 vs. 1.52 ± 0.21, *p* < 0.001).

**Table 3:** Laboratory and imaging biomarkers of the study groups.

**Variable**	**Control Group (*n* = 156)**	**Cryptogenic Stroke (*n* = 52)**	***p-value***
Hemoglobin (g/dL)	13.08 ± 1.87	13.52 ± 1.89	0.083
Platelets (103/μL)	258.34 ± 76.21	245.67 ± 69.83	0.198
Neutrophil (103/μL)	4.97 ± 2.14	5.43 ± 2.38	0.142
Lymphocyte(103/μL)	2.09 ± 0.82	2.24 ± 0.77	0.214
Glucose (mg/dL)	102.31 ± 25.89	107.65 ± 26.12	0.163
Total cholesterol (mg/dL)	186.75 ± 46.32	203.18 ± 48.57	0.009^*^
LDL (mg/dL)	110.92 ± 34.87	119.43 ± 37.25	0.087
HDL (mg/dL)	51.27 ± 13.95	46.83 ± 12.64	0.023^*^
Triglycerides (mg/dL)	131.46 ± 68.37	161.82 ± 72.95	< 0.001^*^
GFR (mL/min/1.73 m^2^)	86.73 ± 22.45	93.15 ± 19.82	0.028^*^
ROPE Score	4.82 ± 2.07	6.39 ± 1.81	< 0.001^*^
ALPS Index	1.52 ± 0.21	1.31 ± 0.18	< 0.001^*^

LDL, low-density lipoprotein; HDL, high-density lipoprotein; GFR, glomerular filtration rate; ROPE, risk of paradoxical embolism. ^*^p < 0.05 indicates statistical significance.

### Univariate and multivariate logistic regression analyses

Univariate logistic regression analyses identified several variables associated with cryptogenic stroke among PFO patients (Table 4). In the univariate analysis, current smoking (OR = 1.672, 95% CI: 1.483–1.792, *p* = 0.028), lower ALPS index (OR = 0.187, 95% CI: 0.091–0.354, *p* < 0.001), higher ROPE score (OR = 1.773, 95% CI: 1.654–1.891, *p* = 0.005), elevated triglyceride levels (OR = 1.841, 95% CI: 1.723–1.952, *p* = 0.009), and higher glucose levels (OR = 1.618, 95% CI: 1.405–1.731, *p* = 0.042) were significantly associated with an increased risk of cryptogenic stroke. Left ventricular ejection fraction (LV EF) was also positively associated (OR = 1.725, 95% CI: 1.612–1.863, *p* = 0.017). In the multivariate logistic regression analysis, a lower ALPS index (OR = 0.126, 95% CI: 0.059–0.273, *p* < 0.001) and elevated triglyceride levels (OR = 1.719, 95% CI: 1.602–1.835, *p* = 0.008) remained independently associated with cryptogenic stroke after adjusting for potential confounders. Left ventricular ejection fraction (LV EF) also showed a statistical association (OR = 1.682, 95% CI: 1.573–1.794, *p* = 0.013); however, given the biological implausibility of this directionality and the possibility of residual confounding or collinearity, this finding should be interpreted with caution and not regarded as evidence of a causal relationship.

**Table 4:** Univariate and multivariate logistic regression analysis for cryptogenic stroke prediction.

**Variables**	**Univariate analysis**		**Multivariate analysis** ^*^	
	**OR (95% CI)**	***p-value***	**OR (95% CI)**	***p-value***
Age	1.512 (0.687–1.625)	0.312	–	–
Diabetes mellitus	1.428 (0.502–1.538)	0.351	–	–
Current smoking	1.672 (1.483–1.792)	0.028	1.497 (0.693–1.608)	0.254
Sex	1.387 (0.491–1.496)	0.382	–	–
Hyperlipidemia	1.583 (0.572–1.702)	0.231		
LV EF (%)	1.725 (1.612–1.863)	0.017	1.682 (1.573–1.794)	0.013
ISA	1.462 (0.423–1.573)	0.394		
PFO diameter (mm)	1.295 (0.845–1.487)	0.156		
Hemoglobin (g/dL)	1.529 (0.754–1.642)	0.198	–	–
Total cholesterol (mg/dL)	1.376 (0.612–1.498)	0.275	–	–
Triglycerides (mg/dL)	1.841 (1.723–1.952)	0.009	1.719 (1.602–1.835)	0.008
Glucose (mg/dL)	1.618 (1.405–1.731)	0.042	1.248 (0.453–1.359)	0.368
ROPE Score	1.773 (1.654–1.891)	0.005	1.556 (0.812–1.667)	0.147
ALPS Index	0.187 (0.091–0.354)	< 0.001	0.126 (0.059–0.273)	< 0.001
GFR (mL/min/1.73 m^2^)	1.331 (0.487–1.442)	0.324		

LV EF, left ventricular ejection fraction; ISA, interatrial septal aneurysm; ROPE, risk of paradoxical embolism; GFR, glomerular filtration rate.

### Association between ALPS index and echocardiographic characteristics

Linear regression analyses were conducted to evaluate the association between ALPS index values and echocardiographic features among PFO patients (Table 5). In the unadjusted Model 1, PFO tunnel length was significantly negatively associated with ALPS index (β = −0.007, 95% CI: −0.012 to −0.002, *p* = 0.004), as was PFO tunnel diameter (β = −0.015, 95% CI: −0.025 to −0.005, *p* = 0.008). After adjustment for age, sex, and BMI in Model 2, the negative association between tunnel length and ALPS index remained significant (β = −0.017, 95% CI: −0.028 to −0.006, *p* = 0.003), whereas the association for tunnel diameter showed a marginal trend (β = −0.024, 95% CI: −0.048 to 0.000, *p* = 0.052). In the fully adjusted Model 3, which accounted for age, sex, BMI, systolic blood pressure, diastolic blood pressure, and education duration, the inverse association between tunnel length and ALPS index persisted (β = −0.024, 95% CI: −0.041 to −0.007, *p* = 0.006), while the association between tunnel diameter and ALPS index was no longer significant (β = −0.156, 95% CI: −0.744 to 0.432, *p* = 0.603).

**Table 5:** Linear regression analysis between ALPS levels and echocardiographic findings among PFO patients.

	**Model 1**		**Model 2**		**Model 3**	
	β **(95% CI)**	***p-values***	β **(95% CI)**	***p-values***	β **(95% CI)**	***p-values***
PFO tunnel length (mm)	−0.007 (−0.012, −0.002)	0.004	−0.017 (−0.028, −0.006)	0.003	−0.024 (−0.041, −0.007)	0.006
PFO tunnel diameter (mm)	−0.015 (−0.025, −0.005)	**0.008**	−0.024 (−0.048, 0.000)	0.052	−0.156 (−0.744, 0.432)	0.603

Model 1, unadjusted model; Model 2, adjusted for age, sex, BMI; Model 3, adjusted for age, sex, BMI, Systolic blood pressure, diastolic blood pressure, and education duration.

Bold values indicate statistically significant results, representing positive/meaningful findings (^*^p < 0.05).

## Diagnostic performance of ALPS index

**Figure 1:**
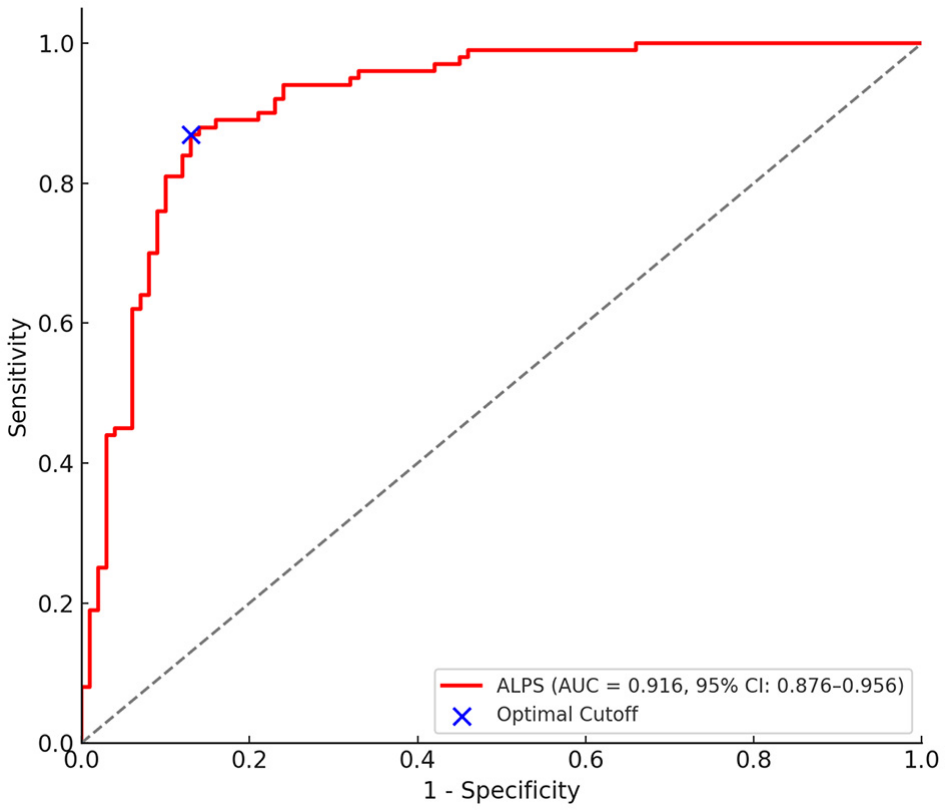
Diagnostic performance of the ALPS index in predicting cryptogenic stroke.

The ROC curve was used to evaluate the diagnostic performance of the ALPS index in predicting cryptogenic stroke among patients with PFO (Figure 1). The results demonstrated a high discriminative ability, with an AUC of 0.916 (95% CI: 0.876–0.956). At the optimal cut-off value, the ALPS index yielded a sensitivity of 90.7% and a specificity of 82.2% for identifying cryptogenic stroke, suggesting that the ALPS index may serve as a reliable imaging biomarker for early detection and risk stratification in PFO-related cryptogenic stroke.

## Proposed mechanisms linking patent foramen ovale to cryptogenic stroke via glymphatic dysfunction

**Figure 2:**
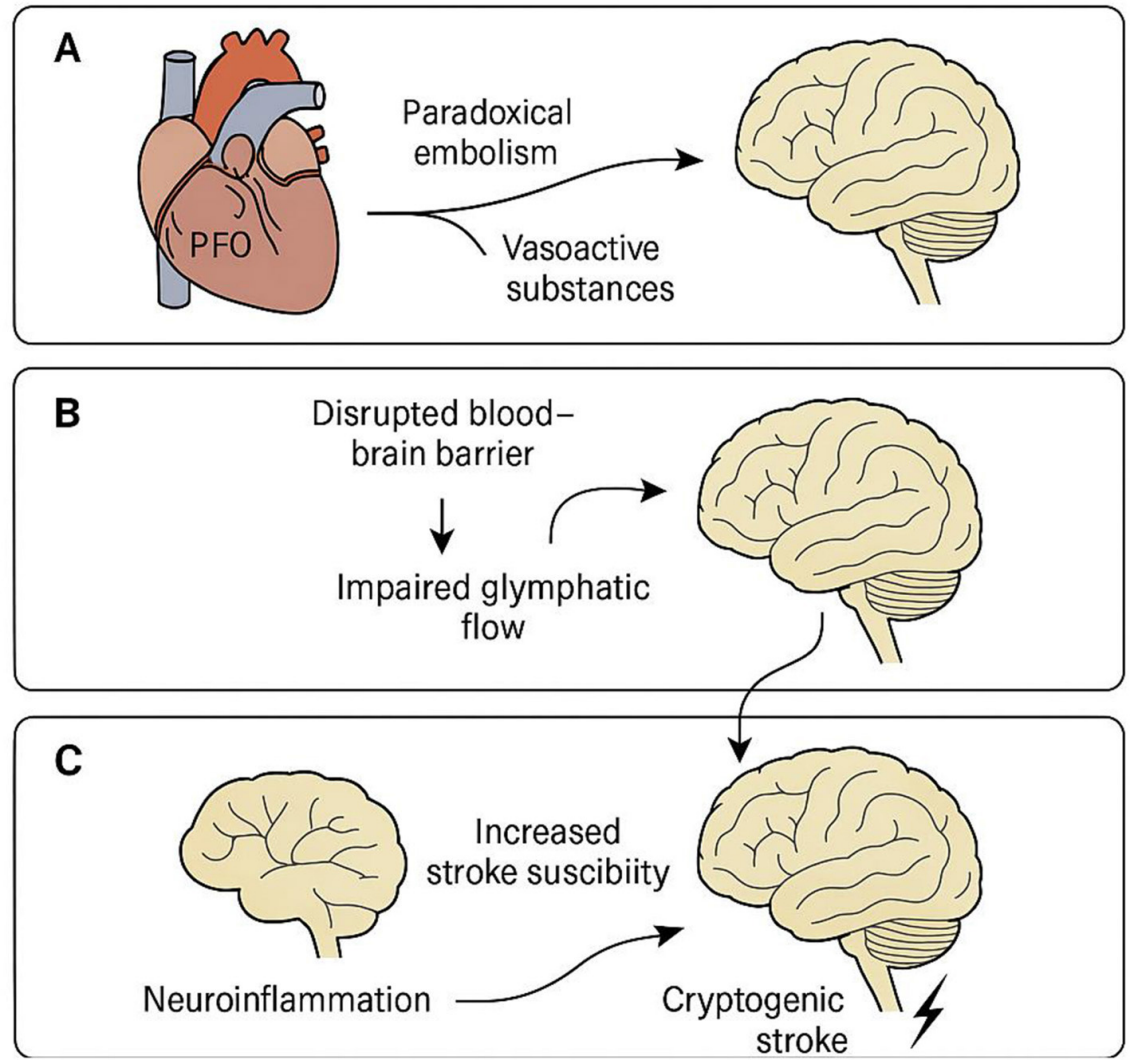
Proposed mechanisms linking patent foramen ovale to cryptogenic stroke via glymphatic dysfunction. Schematic representation of the hypothesized pathophysiological pathways by which a patent foramen ovale (PFO) may contribute to cryptogenic stroke through glymphatic system dysfunction. **(A)** Paradoxical embolism and vasoactive substances (e.g., serotonin, CGRP) bypass pulmonary filtration via PFO and enter cerebral circulation. **(B)** These circulating factors disrupt the blood–brain barrier and impair perivascular cerebrospinal fluid (CSF) flow, leading to reduced glymphatic clearance. **(C)** Glymphatic dysfunction promotes neuroinflammation and the accumulation of neurotoxic metabolites, ultimately increasing susceptibility to ischemic injury and stroke. This central illustration highlights the interaction between embolic and clearance-based mechanisms in the pathogenesis of PFO-related cryptogenic stroke.

A schematic illustration summarizing the hypothesized mechanisms linking PFO to cryptogenic stroke is presented in Figure 2. Panel A depicts paradoxical embolism and the systemic bypass of vasoactive substances via a patent foramen ovale, allowing them to directly enter the cerebral circulation. Panel B illustrates how these embolic or biochemical insults may disrupt the blood–brain barrier and impair glymphatic flow by affecting perivascular clearance. In Panel C, downstream consequences of glymphatic dysfunction—including neuroinflammation, metabolite accumulation, and increased vulnerability to ischemia—are shown to collectively raise the risk of cryptogenic stroke. This illustration highlights the convergence of embolic and clearance failure mechanisms in PFO-related cerebrovascular events.

### Sensitivity and stratified analyses

In age-restricted analyses (≤55 years and ≤ 60 years), the association between lower ALPS index and cryptogenic stroke was directionally consistent with the primary model. In stratified analyses, the ALPS–stroke association was observed across shunt-grade strata and by ISA status. Formal tests for interaction by shunt grade and by ISA did not indicate strong statistical evidence of effect modification. Full estimates are provided in Supplementary Tables S1,S2.

## Discussion

In this study, we found that a lower ALPS index, reflecting impaired glymphatic system function, was significantly associated with an increased risk of cryptogenic stroke among PFO patients. The ALPS index demonstrated strong diagnostic performance, with an AUC of 0.916, and remained an independent predictor of cryptogenic stroke after adjustment for clinical confounders. Additionally, longer PFO tunnel length was independently correlated with lower ALPS index values, suggesting a potential link between structural cardiac anomalies and glymphatic dysfunction.

Interestingly, LV EF showed a statistical association with cryptogenic stroke in our model. However, this finding is counterintuitive from a biological standpoint, as higher ejection fraction is generally protective. We consider this association to be spurious, likely reflecting residual confounding or collinearity with other clinical variables, rather than a true causal effect. Thus, it should be interpreted with caution and requires validation in larger, independent cohorts.

Epidemiological studies have shown that the prevalence of PFO is ~61% among patients with cryptogenic stroke, compared to 19% in patients with ischemic stroke of determined etiology (42). Consistently, in the present study, we found that the prevalence of cryptogenic stroke among PFO patients was 25% (52 out of 208 cases), further supporting the strong association between PFO and cryptogenic stroke. Several mechanisms have been extensively studied in the pathogenesis of PFO-related stroke, including paradoxical embolism, *in situ* thrombus formation, atrial structural and hemodynamic abnormalities, and the presence of interatrial septal aneurysm (43, 44). Furthermore, in patients with a history of cryptogenic vascular events, randomized trials have demonstrated that percutaneous PFO closure significantly reduces the risk of recurrent stroke and transient ischemic attack compared to medical therapy alone (45). Percutaneous closure has been shown to be superior to medical therapy in preventing stroke recurrence (46, 47).

Fluid-attenuated inversion recovery sequences were used to identify stroke lesions and other brain abnormalities (30, 48). DTI-ALPS a technique based on DTI, was employed as a non-invasive method to evaluate glymphatic system activity, which had been demonstrated good stability and excellent interobserver reliability (49–51). Previous studies have demonstrated that PFO is associated with an increased burden of EPVS, reflecting potential glymphatic dysfunction (32). Moreover, structural and hemodynamic abnormalities related to PFO have been implicated in cerebral ischemia and impaired waste clearance (32, 33). Similarly, in our study, we found that patients with PFO had significantly lower ALPS index values compared to controls, indicating impaired glymphatic system function. Furthermore, the lower ALPS index was independently associated with an increased risk of cryptogenic stroke. On the other hand, glymphatic impairment has been increasingly implicated in the pathophysiology of stroke (52, 53). In subarachnoid hemorrhage models, fibrinogen and other blood components have been observed accumulating in enlarged perivascular spaces, accompanied by reduced perivascular AQP4 expression and marked neuroinflammatory responses (54–56). Research further revealed impaired glymphatic inflow and delayed contrast clearance compared to controls (57), along with increased total and phosphorylated tau levels, suggesting compromised waste clearance (58). Restoration of perivascular flow with tissue-type plasminogen activator partially improved glymphatic function (59). Similarly, ischemic stroke models show perivascular space dilation, loss of AQP4 polarity, and astrocyte activation around infarcts (60). These findings collectively suggest that glymphatic dysfunction may play a critical role in mediating brain injury and impaired recovery following both hemorrhagic and ischemic stroke.

We found that impaired glymphatic function, as indicated by a reduced ALPS index, was identified as an independent risk factor for cryptogenic stroke in patients with PFO. Multiple mechanisms may underlie the increased stroke risk in PFO patients. The paradoxical embolism hypothesis proposes that venous thrombi bypass pulmonary filtration and enter cerebral circulation via the PFO, leading to focal ischemia and blood–brain barrier disruption. This process can independently initiate or aggravate glymphatic dysfunction (61–64). In contrast, a neuroinflammatory and clearance dysfunction model posits that hemodynamic disturbances, hypoxia, and unfiltered vasoactive substances (e.g., serotonin, CGRP) may disrupt CSF–interstitial fluid exchange, compromise glymphatic flow, and promote accumulation of neurotoxic metabolites (65–67). Third, vasoactive and inflammatory substances, such as serotonin (5-HT) and calcitonin gene-related peptide (CGRP), which would normally be filtered by the pulmonary circulation, may directly access the cerebral vasculature, promoting neuroinflammation and vascular injury (68–70). Fourth, hemodynamic alterations associated with PFO may increase circulatory resistance, facilitate microthrombus formation, and aggravate BBB damage (71). These pathways may be synergistic, with PDE triggering ischemia while glymphatic failure impairs post-injury clearance, ultimately amplifying stroke risk (72–74). Although the ALPS index demonstrated strong discriminative performance in our cohort, this finding may partly reflect overfitting. External validation in larger, independent, multicenter datasets is required to confirm its robustness and generalizability.

In our cohort, ISA was significantly more common in patients with cryptogenic stroke compared to controls (55.7% vs. 30.1%), consistent with its established role as a marker of high-risk PFO. However, ISA did not retain statistical significance in the multivariate regression analysis. This discrepancy may reflect model instability given the modest sample size, and possible collinearity between ISA, PFO structural parameters, and the ALPS index. It is plausible that ISA contributes to stroke risk primarily through embolic pathways, whereas ALPS index captures the clearance-related dimension, leading to partial overlap in explanatory power. Thus, ISA should still be considered a clinically important risk factor, even though it did not emerge as an independent predictor in our adjusted model.

Beyond mechanistic insights, the clinical application of the ALPS index may offer valuable utility in risk stratification. As a non-invasive biomarker of glymphatic dysfunction, the ALPS index could help identify PFO patients at elevated risk of stroke who might benefit from more aggressive management, including early consideration of percutaneous closure. Conversely, patients with preserved glymphatic function might be suitable for conservative monitoring. Integrating ALPS index measurements into clinical workflows could thus complement traditional assessments (e.g., ROPE score, shunt grade) to refine individualized decision-making for stroke prevention.

Our findings support an association between impaired glymphatic function, as reflected by a lower ALPS index, and stroke occurrence. However, given the cross-sectional design, causality cannot be inferred. It remains unclear whether glymphatic dysfunction predisposes to stroke, or instead represents a consequence of ischemic injury (e.g., AQP4 polarity loss, EPVS dilation). Therefore, longitudinal studies with repeated neuroimaging assessments are essential to clarify temporal directionality. Moreover, prospective validation in larger and more diverse populations is required to determine whether ALPS index can serve as a reliable biomarker for stroke risk stratification.

## Study limitations and future direction

Several limitations of this study should be acknowledged. First, the cross-sectional design restricts causal inference. Although a significant association between lower ALPS index and cryptogenic stroke was observed, it remains unclear whether glymphatic dysfunction is a cause or consequence of stroke. Prospective cohort studies or interventional investigations, such as evaluating ALPS index changes after PFO closure, are needed to clarify this relationship. Second, potential selection bias may exist. This was a single-center study, which may limit the generalizability of the findings to broader PFO populations across different regions and ethnicities. In addition, the control group consisted of PFO patients without a history of stroke rather than healthy individuals, which may have led to an underestimation of effect sizes and a shift in baseline ALPS index values. Future studies including healthy controls are warranted to validate and extend our findings. Additionally, the control group consisted of PFO patients without a history of stroke rather than healthy individuals, which may have led to an underestimation of the degree of ALPS index abnormalities. Third, there were methodological limitations related to imaging. While DTI-ALPS is a useful indirect marker of glymphatic function, it does not directly quantify CSF–interstitial fluid exchange rates. Future studies combining DTI-ALPS with advanced imaging modalities, such as dynamic contrast-enhanced MRI or molecular PET imaging, could provide more direct assessments. Fourth, unmeasured confounders may have influenced the results. For example, sleep quality and obstructive sleep apnea (OSA)—both major modulators of glymphatic flow—were not assessed in this study. Furthermore, the use of medications such as statins and antihypertensives, which could influence lipid metabolism, vascular function, and possibly ALPS index values, was not systematically recorded. Finally, microembolic signals (MES) on transcranial Doppler were not measured, which could have provided valuable mechanistic information to help disentangle embolic from clearance-related pathways. Future studies should incorporate these parameters to enhance the mechanistic and clinical interpretability of ALPS index findings in PFO-related cryptogenic stroke. Additionally, the unexpected positive association between LV EF and cryptogenic stroke may reflect collinearity or residual confounding, and should not be considered as evidence of a causal relationship. Finally, challenges remain regarding clinical translation. The optimal cut-off value for the ALPS index requires validation in larger, multicenter cohorts, and its relationship with stroke recurrence and long-term outcomes was not assessed in this study. Future directions include developing AI-assisted fully automated ALPS index analysis pipelines to enhance reproducibility and integrating 7T MRI and Q-space imaging techniques to achieve submillimeter visualization of glymphatic pathways. Clinical applications may also explore ALPS index-guided PFO management strategies, with more aggressive interventions in patients with impaired glymphatic function.

## Conclusions

In summary, among PFO patients, impaired glymphatic function, as indicated by a lower ALPS index, was independently associated with an increased risk of cryptogenic stroke. The ALPS index demonstrated strong discriminative performance in this cohort and may represent a potential imaging biomarker for stroke risk stratification. However, these findings are preliminary and require validation in larger, independent cohorts before translation into clinical practice. These findings highlight the interplay between structural cardiac anomalies and central nervous system clearance dysfunction, offering new insights into the pathophysiology of cryptogenic stroke and potential targets for early risk assessment and intervention.

## Data Availability

The raw data supporting the conclusions of this article will be made available by the authors, without undue reservation.
