# Supplementary material for: Association between glymphatic dysfunction and cryptogenic stroke risk in patients with patent foramen ovale: a retrospective cross-sectional study
Source: Front Neurol. 2025 Sep 22;16:1620772. doi: 10.3389/fneur.2025.1620772 (PMC12499356; doi:10.3389/fneur.2025.1620772)
Supplement: Supplementary file 1 [file Table_1.docx]

**Supplementary Table S1. Multivariable models in age-restricted subsets**

**(A) Age ≤55 years**

| **Variable** | **OR (95% CI)** | **P-value** |
| --- | --- | --- |
| **ALPS index (per 0.10 ↑)** | **0.76 (0.64–0.90)** | **0.001** |
| Triglycerides (per 10 mg/dL ↑) | 1.08 (1.02–1.15) | 0.010 |
| HDL-C (per 10 mg/dL ↑) | 0.88 (0.69–1.12) | 0.298 |
| Hypertension | 1.42 (0.77–2.64) | 0.258 |
| Current smoking | 1.51 (0.86–2.66) | 0.151 |
| ROPE score (per 1-point ↑) | 1.07 (0.93–1.22) | 0.352 |
| LVEF (per 5% ↑) | 1.10 (0.92–1.32) | 0.300 |
| PFO tunnel length (per 1 mm ↑) | 1.03 (0.96–1.12) | 0.392 |
| PFO tunnel diameter (per 1 mm ↑) | 1.18 (0.90–1.55) | 0.230 |
| ISA (present) | 1.29 (0.73–2.26) | 0.383 |
| Age (per 1 year ↑) | 1.02 (0.99–1.05) | 0.208 |
| Sex (male) | 1.21 (0.70–2.10) | 0.490 |
| BMI (per 1 kg/m² ↑) | 1.03 (0.98–1.09) | 0.238 |

**(B) Age ≤60 years**

| **Variable** | **OR (95% CI)** | **P-value** |
| --- | --- | --- |
| **ALPS index (per 0.10 ↑)** | **0.79 (0.69–0.91)** | **0.002** |
| Triglycerides (per 10 mg/dL ↑) | 1.09 (1.03–1.15) | 0.004 |
| HDL-C (per 10 mg/dL ↑) | 0.93 (0.77–1.12) | 0.438 |
| Hypertension | 1.36 (0.82–2.25) | 0.235 |
| Current smoking | 1.47 (0.91–2.37) | 0.115 |
| ROPE score (per 1-point ↑) | 1.05 (0.94–1.17) | 0.398 |
| LVEF (per 5% ↑) | 1.12 (0.96–1.31) | 0.162 |
| PFO tunnel length (per 1 mm ↑) | 1.04 (0.98–1.10) | 0.205 |
| PFO tunnel diameter (per 1 mm ↑) | 1.16 (0.94–1.44) | 0.170 |
| ISA (present) | 1.24 (0.78–1.98) | 0.358 |
| Age (per 1 year ↑) | 1.01 (0.99–1.03) | 0.280 |
| Sex (male) | 1.18 (0.74–1.89) | 0.486 |
| BMI (per 1 kg/m² ↑) | 1.03 (0.99–1.07) | 0.106 |

**Supplementary Table S2. Stratum-specific association between ALPS and stroke by shunt grade (ISAC)**

| **Shunt grade (ISAC)** | **N (stroke/total)** | **OR for ALPS (per 0.10 ↑)** | **95% CI** | **P-value** |
| --- | --- | --- | --- | --- |
| Grade 1 | 12/68 | 0.84 | 0.70–1.02 | 0.080 |
| Grade 2 | 19/78 | **0.78** | **0.66–0.92** | **0.003** |
| Grade 3 | 21/62 | **0.73** | **0.60–0.89** | **0.001** |

**Interaction (ALPS × shunt grade)**

- Wald: χ² = 3.8, df = 2, **P = 0.15**
- LRT: Δχ² = 4.0, df = 2, **P = 0.14**

**Supplementary Table S3. Stratum-specific association between ALPS and stroke by ISA**

| **ISA status** | **N (stroke/total)** | **OR for ALPS (per 0.10 ↑)** | **95% CI** | **P-value** |
| --- | --- | --- | --- | --- |
| Absent | 23/137 | **0.80** | **0.69–0.94** | **0.006** |
| Present | 29/71 | **0.76** | **0.63–0.92** | **0.004** |

**Interaction (ALPS × ISA)**

- Wald: χ² = 0.26, df = 1, **P = 0.61**
- LRT: Δχ² = 0.27, df = 1, **P = 0.60**

**Supplementary Table S4. Model Diagnostics of Multivariable Logistic Regression**

| **Covariate** | **VIF** | **Tolerance** |
| --- | --- | --- |
| Age | 1.32 | 0.76 |
| Hypertension | 1.41 | 0.71 |
| Dyslipidemia | 1.28 | 0.78 |
| Smoking status | 1.22 | 0.82 |
| LV ejection fraction | 1.35 | 0.74 |
| Triglycerides | 1.47 | 0.68 |
| ALPS index | 1.51 | 0.66 |

**Hosmer–Lemeshow Goodness-of-Fit Test:**

- χ² = 6.47, df = 8, P = 0.595 (indicating good fit).

**Nagelkerke R²:** 0.41

**Area under ROC (cross-validated):** 0.82
